# Effects of Combining Exercise and Dietary Shifts on Motor Coordination and Oxidative Markers in a High‐Fat Diet Model in Rats

**DOI:** 10.1002/cph4.70074

**Published:** 2025-11-25

**Authors:** Manuel Jiménez‐García, Maria Magdalena Quetglas‐Llabrés, Maria del Mar Ribas‐Taberner, Antoni Sureda‐Gomila, David Moranta‐Mesquida, Silvia Tejada‐Gavela

**Affiliations:** ^1^ Group of Neurophysiology, Department of Biology University of the Balearic Islands Palma de Mallorca Balearic Islands Spain; ^2^ Group of Neurophysiology, Behavioural Studies and Biomarkers Health Research Institute of the Balearic Islands (IdISBa) Palma Spain; ^3^ Research Group on Community Nutrition and Oxidative Stress University of the Balearic Islands Palma de Mallorca Balearic Islands Spain; ^4^ CIBEROBN (Physiopathology of Obesity and Nutrition) Palma de Mallorca Balearic Islands Spain; ^5^ Group of Community Nutrition and Oxidative Stress (NUCOX) Health Research Institute of the Balearic Islands (IdISBa) Palma Spain

**Keywords:** fatty liver, glucose, irisin, oxidative stress, rotarod test

## Abstract

Metabolic Associated Fatty Liver Disease (MAFLD) is a prevalent chronic condition with limited therapeutic options, making lifestyle interventions a primary strategy. This study investigated whether exercise, alone or with dietary modifications, mitigates HFD‐induced alterations in rats of both sexes. The motor coordination, plasma glucose and irisin levels, oxidative stress and inflammatory biomarkers (catalase, superoxide dismutase, glutathione peroxidase, malondialdehyde and myeloperoxidase) in liver and muscle, and hepatic Nrf2, NF‐κB, and UCP‐2 expression were evaluated. Rats were fed a HFD for 3 months, followed by 2 months of interventions consisting of exercise and a shift to a standard diet (SD) or antioxidant‐rich diet. Control and HFD groups received pellet and HFD, respectively, for the full 5 months. The results showed improvements in weight gain, motor performance, and antioxidant profiles in tissues when exercise was combined with dietary changes in both sexes. Exercise alone was enough to improve motor coordination in males. Plasma glucose and irisin recovered control values, especially when exercise was combined with a healthier diet in both sexes. Nrf2, NF‐κB and UCP‐2 expression, altered by the HFD, were also restored after interventions. Overall, the combination of exercise and an antioxidant‐rich diet produced the most pronounced improvements across all parameters. In conclusion, combining exercise and a healthier diet, especially rich in antioxidants, led to marked improvements in motor function and plasma and tissue biomarkers with slight differences between females and males.

## Graphical abstract

Combining exercise with a healthier diet improved weight gain, motor coordination, and antioxidant profiles in liver and muscle tissues in a high‐fat diet rat model of Metabolic Associated Fatty Liver Disease (MAFLD). These interventions restored molecular markers, highlighting coordinated tissue benefits and their potential in managing MAFLD in both sexes.

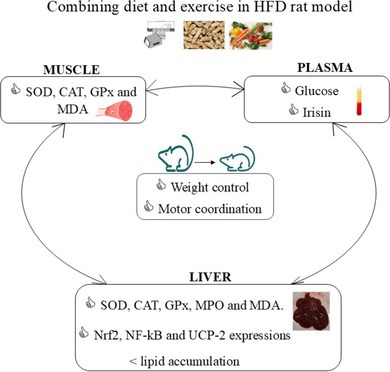

## 1 Introduction

Currently, many diseases are underdiagnosed, including non‐alcoholic fatty liver disease (NAFLD) (Alexander et al. 2018; Nielsen et al. 2022). This disease, also known as metabolic dysfunction‐associated fatty liver disease (MAFLD) to improve classification and better encompass its diverse manifestations (Eslam et al. 2020), is characterized by the accumulation of fat in liver cells (Ntona et al. 2023). The prevalence of this disease affects both sexes and has been increasing over decades, largely due to western dietary habits and increasingly sedentary lifestyles (Yahoo et al. 2023). It is estimated that 32% of adults around the world suffer from MAFLD, with higher prevalence in North Africa and the Middle East (43%). North America and Australia are close with a prevalence of 39%, followed by Europe with 32%. The lowest rates are observed in Southeast Asia (24%) (Younossi et al. 2023). Moreover, males show a higher prevalence (close to 5 times more) than women under 50 years old (Cheng et al. 2024; Zhou et al. 2007). MAFLD is a liver disease that affects not only the liver but also extrahepatic organs and systems at different levels. The accumulation of lipids in hepatocytes, mainly triglycerides, cholesterol, and free fatty acids, induces insulin resistance (Nassir 2022), leading to increased blood sugar and lipid levels (Chao et al. 2019). This lipid accumulation also contributes to oxidative stress and inflammation, favoring disease progression from simple steatosis to cirrhosis or even hepatocarcinoma (Friedman et al. 2018). The alterations caused by MAFLD increase the risk of developing other diseases, such as diabetes (Tanase et al. 2020), heart disease (Kasper et al. 2021), strokes (Hadjihambi 2022), and cognitive decline (Colognesi et al. 2020). Lipid accumulation also occurs in peripheral tissues, such as adipose tissue and skeletal muscle, where it can impair metabolic function contributing to insulin resistance and hyperinsulinemia and exacerbating systemic inflammation (Altajar and Baffy 2020; Jimenez‐Gutierrez et al. 2022; Sandireddy et al. 2024; Zheng et al. 2024). Given its key role in metabolic regulation, alterations in skeletal muscle function can actively drive the development and progression of metabolic disorders like MAFLD (Mihailovic et al. 2021; Xiao et al. 2022).

Oxidative stress induced by a high‐fat diet has also been described as a major contributor to insulin resistance, inflammation, and cellular damage (Matsuzawa‐Nagata et al. 2008; Yuzefovych et al. 2013). Thus, enzymes like superoxide dismutase (SOD), catalase (CAT), glutathione peroxidase (GPx), and myeloperoxidase (MPO), as well as the lipid damage marker malondialdehyde (MDA) levels, can be altered by a high‐fat diet (HFD) (Eleazu et al. 2021; Piek et al. 2019). Antioxidant enzymes (SOD, CAT, and GPx) neutralize reactive oxygen species (ROS) and lipid peroxides, protecting cells from oxidative damage and inflammation. On the other hand, MPO, an enzyme mainly present in neutrophils, participates in the production of ROS since it catalyzes the formation of hypochlorous acid (HOCl), from hydrogen peroxide (H_2_O_2_) and chloride (Amanzada et al. 2011). The release of MPO from neutrophils is increased under pro‐inflammatory conditions, such as MAFLD, and may promote endothelial dysfunction and the development of metabolic complications by enhancing the oxidation of lipids and lipoproteins (Piek et al. 2019). MDA, a by‐product of lipid peroxidation, is elevated in MAFLD correlating with disease severity, and reflecting increased oxidative stress and liver injury (Eleazu et al. 2021). Aside from the mechanisms previously described, diseases like MAFLD also affect motor coordination. Animal studies have shown that a high‐lipid and high‐sugar diet or HFD impairs motor coordination and cognitive function with deficits in exploring or recognizing new objects (Kumar et al. 2023; Singh et al. 2021; Veniaminova et al. 2020). Regular exercise has been reported to counteract some of these detrimental effects. In this sense, irisin, a 112‐amino acid myokine released by skeletal muscle during physical exercise (Ma et al. 2021), exerts protective effects on liver metabolism by promoting lipid oxidation and reducing hepatic steatosis inflammation and fibrosis through the activation of transcription pathways, such as Nrf2 (Nuclear Factor Erythroid 2‐Related Factor 2) and inhibiting NF‐κβ (Nuclear Factor kappa‐light‐chain‐enhancer of activated B cells) (Zhu et al. 2021).

Altogether, dietary changes and exercise should be considered pivotal to mitigating the effects induced by HFD. Some human studies have evidenced that diets rich in bioactive compounds help counteract the detrimental effects of HFD (Shabani et al. 2024; Zhang et al. 2022; Zhu et al. 2023), mainly due to the anti‐inflammatory and antioxidant properties (such as polyphenols that are abundant in fruits and vegetables) (Quetglas‐Llabres et al. 2024) ameliorating oxidative stress and pro‐inflammatory markers and, consequently, reducing cellular damage (Almoraie and Shatwan 2024). Moreover, antioxidants have been shown to provide potential benefits for motor coordination and cognitive function through their effects on reducing oxidative stress and brain health by decreasing neuroinflammation, enhancing neurogenesis, and supporting cerebrovascular function (Gabbia et al. 2022). The current study aims to evaluate how the combination of diet and exercise could help counteract the harmful effects of a HFD in both male and female rats by assessing motor coordination and oxidative markers in the liver and muscle.

## 2 Materials and Methods

### 2.1 Animals and Treatments

Wistar rats (35 males and 35 females), aged 5 months, were individually housed in standard cages. They were kept under controlled environmental conditions, including a temperature of 20°C ± 2°C, 70% relative humidity, and a 12‐h light/dark cycle, with lights turning on at 08:00. Every effort was made to minimize the number of animals used and to reduce their suffering. All experimental procedures adhered to the EU Directive 2010/63/EU, the Spanish Royal Decree 53/2013 concerning the protection of vertebrate animals used for scientific purposes, and the ethical guidelines established by the Bioethical Committee of the University of the Balearic Islands (2023/02/AEXP).

**FIGURE 1:**
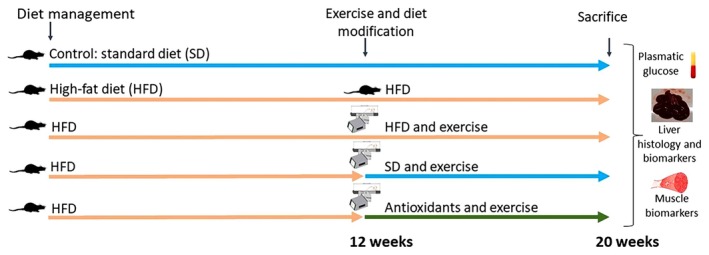
Experimental design (20 weeks). The dietary intervention and the performance of exercise were initiated at Week 12 for three of the original HFD groups. The Rotarod test was accomplished at Weeks 12 and 20. *N* = 7 per group and sex. Plasma, liver and gastrocnemius muscle tissue were collected for subsequent analysis.

The animals of both sexes were randomly divided into five groups, each consisting of seven individuals. The animals were weighed throughout the experiment (Figure S1), and weight gain was calculated as the difference between the weight on the day of sacrifice and the initial body weight at the beginning of the experiment. The treatments varied based on diet and exercise protocols (Figure 1). The control group received standard food (Panlab A04, Panlab S.L.U., Barcelona, Spain) that covers the health needs for the entire duration of the experiment (20 weeks). The remaining four groups were provided with a high‐fat diet (HFD) (Sampey et al. 2011), two of which received it for 20 weeks and the other two for three months. During the last two months of the experiment, these two latter groups switched diets: one transitioned to a standard diet (like the control group), and the other was placed on an antioxidant‐rich diet. The HFD included a daily intake of 25–30 g of high‐fat foods, including biscuits, red meat, cheese, and sweets (Sampey et al. 2011). The antioxidant diet provided between 6 and 8 g of broccoli, apple, and banana each, along with approximately 5 g of blueberries. Altogether, this diet amounts to a total of 25–30 g per rat per day. The composition of HFD and antioxidant diet is included in Table 1. Throughout the experiment, all groups had tap water available ad libitum.

**TABLE 1:** Calorie content and total nutrient compositions for the main diets.

Component	HFD	Antioxidant‐rich diet
Energy (kcal)	85.96	14.85
Total protein (g)	1.572	0.391
Total fat (g)	4.314	0.086
Polyunsaturated fatty acids (g)	0.420	0.032
Monounsaturated fatty acids (g)	1.544	0.015
Saturated fatty acids (g)	2.176	0.030
Cholesterol (mg)	11.318	0
Fiber (g)	0.333	0.917
Carbohydrates (g)	10.136	3.055
Water (g)	29.538	24.51

In addition to the dietary factor, three of the groups performed exercise during the last two months of the experiment (Figure 1). The LE8710M Rodent Treadmill (Panlab, Barcelona, Spain) was used for the exercise sessions. This device consists of a treadmill with metal bars at the end, which can deliver a mild electrical current to encourage the rats to keep walking. A 5‐day habituation period was provided. Afterward, the rats exercised 5 days a week, with the speed and duration increasing each week (Table 2), eventually reaching a maximum of 12 min of exercise at a speed of 47.7 cm/s (Salehi et al. 2019).

**TABLE 2:** Details of the characteristics of the exercise sessions performed in the treadmill.

Week	Speed (cm/s)	Time (min)
1	16.9	5
2	20.7	6
3	24.7	7
4	28.5	8
5	32.6	9
6	36.6	10
7	39.8	11
8	47.7	12

After the 20‐week period, the animals were decapitated and blood was directly obtained. A portion of the liver was collected for histological analysis. The remaining liver and gastrocnemius muscle were extracted. Total body fat of every animal was collected and weight (BL2002 Basic (Giorgio Bormac S.r.l., Módena, Italy)), and the adiposity index was calculated as (total body fat/final body weight) × 100. Tissue and plasma samples were rapidly frozen in liquid nitrogen and stored at −80°C until further analysis.

### 2.2 Rotarod Test

To assess motor coordination, the rotarod test (Model LE8350, Panlab, S.L.U, Barcelona, Spain) was performed before implementing the diet and exercise modifications (3 months after the initiation of the HFD induction) and at the end of the experiment, before the sacrifice (5 months after the initiation of the experiments) (Figure 1). This test involves a rotating cylinder that can spin at either a fixed speed or with acceleration (4–40 rpm). Once the animals were placed on the cylinder, the time and speed they achieved without falling off are recorded (Bogo et al. 1981). The rotation and speed were adjusted via an electronic panel located at the front of the device. Four days before the tests, the animals underwent a habituation phase in which the cylinder maintained a constant speed (5 rpm). The goal during this phase was for the rats to remain on the apparatus. On the test day, the cylinder was set to accelerate (4–40 rpm over a 5‐min period). Each rat performed the test five times, with a 5‐min rest period between each trial. For data analysis, the average time and speed achieved by each rat were calculated.

### 2.3 Histology

Medial lobe of the liver was preserved in 10% paraformaldehyde for 24 h to prepare them for histological examination (Jia et al. 2019). Prior to this step, the samples were incubated overnight in a 20% sucrose solution to minimize tissue damage. After rinsing with PBS, they were embedded in a freezing medium (LEICA, Ref: 14020108926) on a glass plate. Tissue sections, 10 μm thick, were cut using a cryostat (Leica CM1520) and placed in PBS to avoid damage. These sections were then mounted on slides for staining. The samples underwent fixation and staining using the Oil Red O method, involving sequential immersion in 10% formalin, 60% isopropanol, Oil Red O solution, 60% isopropanol, distilled water, Mayer's hematoxylin, distilled water, and tap water. The Oil Red O selectively stains lipid deposits, which appear as red spots (Kinkel et al. 2004). Hepatic lipid droplets were quantified systematically to ensure representative and reproducible data. Specifically, analyses were carried out in four independent animals per group, and from each liver four different tissue sections were examined. Within each section, five random fields were selected for image acquisition and quantification. Microscopic images were obtained with a LEICA MZ16, using a 10×/0.25 objective lens. For quantification of acquired images, ImageJ was used to obtain intensities of red pixels and the percentage of stained areas was calculated.

### 2.4 Plasma Analysis

Glucose concentrations in plasma were measured according to the manufacturer's instructions (SPINREACT, S.A.U., Girona, Spain). The assay relies on the measurement of nicotinamide adenine dinucleotide (NADH) production, which correlates directly with glucose levels in the sample. Two reagents were utilized: R1, a buffer solution, and R2, which contains the necessary enzymes, alongside a glucose calibrator. The contents of R2 were mixed with R1, and the resulting working reagent was added to a microplate along with the samples or the standard. The absorbance was read at 340 nm using a spectrophotometer (Bioteck, Epoch), and the equation “([(absorbance)sample−(absorbance)blank]/[(absorbance)standard−(absorbance)blank]) × 100” was used to calculate the glucose concentrations (mg/dL).

Irisin concentrations in plasma were determined using the ELISA kit ER1486 from FineTest (Wuhan, China), based on sandwich immunoassay technology, and following the manufacturer's instructions. Samples and standards were incubated in a 96‐well plate pre‐coated with an antibody specific for irisin. After incubation and washing to remove unbound conjugates, a biotinylated antibody was added to detect irisin, followed by the addition of HRP‐Streptavidin. Finally, a substrate was added to produce a colorimetric signal, which was measured at 450 nm in a microplate reader. The concentration of irisin was calculated based on a standard curve.

### 2.5 Oxidative Biomarkers

A sample dispersing system (Ultra‐Turrax T10 Disperser, IKA, Staufen, Germany) was used to first homogenize the liver and gastrocnemius muscle in a Tris–HCl buffer (10 mM, pH 7.5), followed by centrifugation at 9000 rpm for 10 min at 4°C. The supernatant obtained was used for the biochemical analysis. The activities of superoxide dismutase (SOD), catalase (CAT), glutathione peroxidase (GPx), and myeloperoxidase (MPO) were measured in gastrocnemius muscle homogenates and in hepatic samples following previously described methods (Sureda et al. 2004). SOD activity was determined using a xanthine/xanthine oxidase system that generates the superoxide anion, which reduces cytochrome C, and the absorbance was measured at 550 nm. CAT activity was quantified spectrophotometrically by monitoring the breakdown of H_2_O_2_. GPx activity was assessed by an assay involving H_2_O_2_ and NADPH, tracking the decrease in NADPH absorbance at 340 nm as it was oxidized to NADP^+^. MPO activity was measured by guaiacol oxidation (Capeillere‐Blandin 1998). Protein concentration was determined using a commercial kit (Merck Life Science S.L.U., Madrid, Spain) to normalize the enzyme activity values. All measurements were conducted at 37°C using a Shimadzu UV‐2100 spectrophotometer (Shimadzu Corporation, Kyoto, Japan). As a marker of lipid peroxidation, MDA levels were quantified in hepatic and gastrocnemius muscle samples using a colorimetric assay kit (Merck Life Science S.L.U., Madrid). Briefly, standards or samples were mixed with a 10.3 mM solution of n‐methyl‐2‐phenyl‐indole in acetonitrile (3:1 ratio). After adding 12 N HCl, the mixture was incubated for 1 h at 45°C. Absorbance was recorded at 586 nm, and the MDA concentration was calculated by comparing the sample readings to a standard curve of known MDA concentrations.

### 2.6 Gene Expression

RNA extraction from the liver was performed using Tripure reagent (Tripure Isolation Reagent, Roche Diagnostics, Mannheim, Germany), following the manufacturer's instructions. Subsequently, 1 μg of RNA from each sample was subjected to reverse transcription using TaqMan Reverse Transcription Reagents (Life Technologies, Van Allen Way, Carlsbad, CA, USA) at 42°C for 60 min, followed by 5 min at 99°C, in a final volume of 10 μL. A total of 3 μL of the resulting cDNA was amplified using LightCycler 480 SYBR Green I Master (Roche Diagnostics, Mannheim, Germany). The target cDNAs—Nrf2, NF‐ĸβ, and UCP2—were amplified using rat β‐actin RNA as the reference gene, in a LightCycler 96 for 45 cycles, following an initial denaturation step of 10 min at 95°C. The primers used and amplification conditions are detailed in Table 3. Relative quantification was performed using the 2(^−ΔΔCt^) method, with values normalized to those of the control group.

**TABLE 3:** Primer sequences and conditions used in real‐time PCRs.

Gene	Primer		Conditions
B‐Actin	Fw:	5′‐AGG GAAATCGTGCGTGAC‐3′	95°C 15 s
	Rv:	5′‐CGCTCATTGCCGATAGTC‐3′	60°C 30 s
			72°C 30 s
Nrf2	Fw:	5′‐CTTTCGTAGCCTCCATGAAGCA‐3′	95°C 10 s
	Rv:	5′‐AGTGTCTGGGTCATAGCATTCCA‐3′	60°C 30 s
			72°C 30 s
NFĸβ	Fw:	5′‐ACGATCTGTTTCCCCTCATCT‐3′	95°C 15 s
	Rv:	5′‐TGCTTCTCTCCCCAGGAATA‐3′	57°C 30 s
			72°C 30 s
UCP2	Fw:	5′‐CAATGTTGCCCGAAATGCCA‐3′	95°C 10 s
	Rv:	5′‐TCTTGACCACATCAACGGGG‐3′	60°C 30 s
			72°C 30 s

Abbreviations: B‐actin, beta‐actin; NF‐ĸβ, nuclear factor‐kappa beta; Nrf2, nuclear factor erythroid 2‐related factor 2; UCP2, uncoupling protein‐2.

### 2.7 Statistical Analysis

The statistical analysis was performed using GraphPad Prism version 8.3.0. Data are reported as the mean ± standard error of the mean (SEM). A *p*‐value less than 0.05 was considered statistically significant. The Shapiro–Wilk test was used to assess the normality of the data distribution. A one‐way ANOVA was employed to analyze the data, and if significant differences were found, a post hoc Least Significant Difference (LSD) test was conducted to identify specific group comparisons.

## 3 Results

**FIGURE 2:**
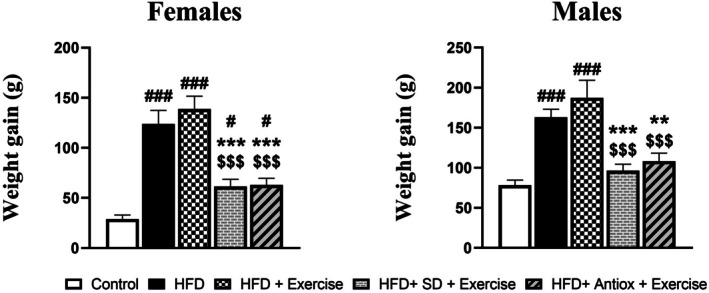
Weight gain (g) of female and male rats (*n* = 7/sex and group) as the difference between the final weight and the initial weight at the beginning of the experiment. The initial weight at the beginning of the experiment for control, HFD, HFD + Exercise, HFD + SD + Exercise, and HFD + Antiox + Exercise groups was for females 28.96 ± 3.95, 123.9 ± 13.57, 138.7 ± 12.86, 61.37 ± 7.16, and 62.94 ± 6.66; and for males 78.31 ± 6.34, 161.1 ± 9.98, 187.5 ± 21.74, 96.33 ± 8.08, and 108.3 ± 10.05, respectively. Data represent mean ± S.E.M. One‐way ANOVA and LSD post hoc. # *p* < 0.05 and ### *p* < 0.001 with respect to control group; **p < 0.01 and ***p < 0.001 with respect to HFD group; $$$ *p* < 0.001 with respect to HFD + Exercise group. Antiox, antioxidant; HFD, high‐fat diet; SD, standard diet.

All groups showed an increase in weight compared to their initial values (Figure 2), but this increase was significantly higher in the HFD and HFD + Exercise groups for both females and males. In both sexes, the HFD and HFD + Exercise groups showed significantly higher values compared to the other three groups. Additionally, in females, the groups that reversed the diet and simultaneously performed exercise also showed significant differences compared to the control group.

**FIGURE 3:**
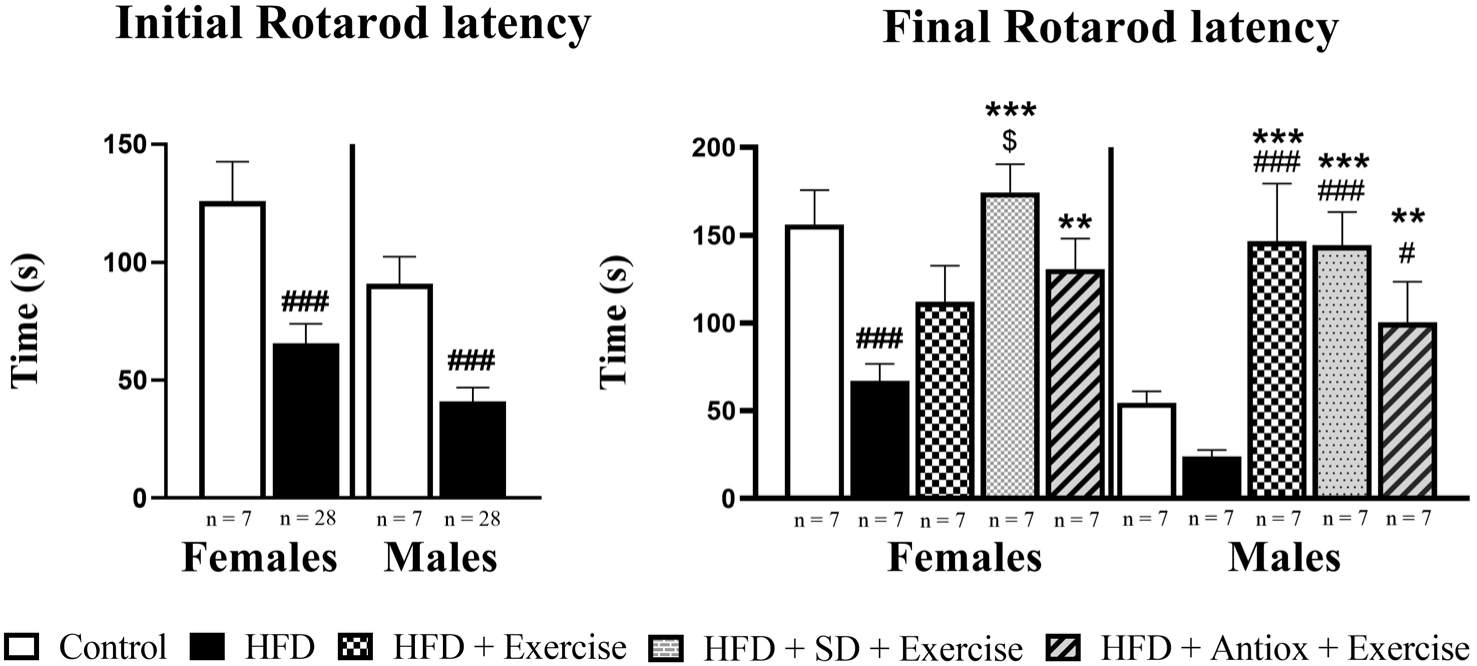
Rotarod test in both sexes (*n* = 7/sex and group): Control, high‐fat diet (HFD), HFD and exercise, and HFD groups with changes to standard (SD) or antioxidant‐rich (Antiox) diets and exercise performance. Initial latency refers to the test performed at 3 3 months after the beginning of the experiment. Final latency refers to the test performed at the end of the study (5 months after the onset). Data represent mean ± S.E.M. One‐way ANOVA and LSD post hoc. # *p* < 0.05 and ### *p* < 0.001 with respect to control group; **p < 0.01 and ***p < 0.001 with respect to HFD group; $ *p* < 0.05 with respect to HFD + Exercise group.

Figure 3 shows the results obtained from the rotarod coordination test. The first graph shows the results after 3 months of control diet and HFD. In both sexes, the control group had better results than the HFD group, since it managed to stay on the rotatory wheel for a longer time. After 3 months, the HFD was divided into different groups for dietary and exercise interventions. After 5 months from the beginning of the experiments, in females, the two groups that followed dietary changes and exercise for two months, along with the control group, displayed higher performance in the test than the HFD and HFD + Exercise groups (although greater permanence was observed in the latter). In males, the three groups that performed exercise showed an increase in performance, with significant differences with respect to the control and HFD groups.

**FIGURE 4:**
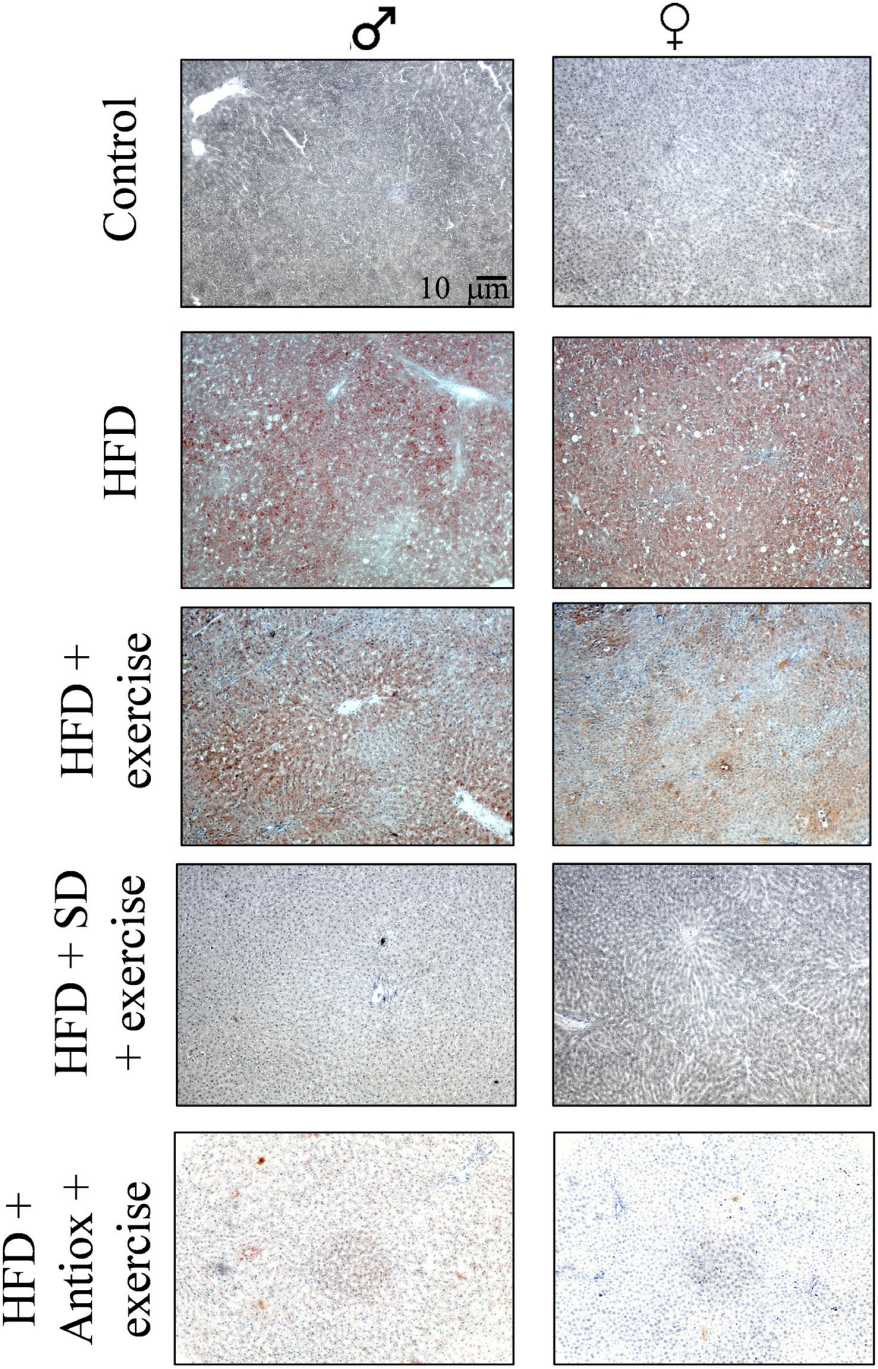
Representative histological liver images for every treatment and sex. Percentages indicate the area stained with the Oil Red O solution. Scale bar = 10 μm. Antiox, antioxidant; HFD, high‐fat diet; SD, standard diet.

Table 4 shows the total body fat and the adiposity index in the different studied groups. The total body fat was higher in the HFD group compared to all the other groups, although the intervention combining exercise and diet change resulted in a higher reduction of the total body fat when compared to the exercise performance. Similarly, the adiposity index was improved after the combination of diet and exercise, but not with only the exercise performance in females. The histological results showed differences in the percentage of lipid droplets in the liver across the different groups stained by Oil Red O solution (Figure 4). There was no lipid accumulation in the hepatic samples of control groups, for both sexes. The lipid accumulation in the HFD group showed the highest percentages for females and males. These values slightly decreased when the animals on a HFD also performed exercise When HFD was changed to a healthier diet (SD and Antiox) together with exercise performance, lipid accumulation was considerably lower, for females and males (Table 4).

**TABLE 4:** Effects of the interventions in the adiposity characteristics of the rats.

Experimental group	Total body fat (g)		Adiposity index		Hepatic lipid accumulation (%)	
	Females	Males	Females	Males	Females	Males
Control	3.49 ± 0.30	7.88 ± 0.97	1.23 ± 0.10	1.56 ± 0.19	0	0
HFD	24.18 ± 3.91***	52.44 ± 3.72***	7.80 ± 0.99	8.33 ± 0.46	9.41	10.54
HFD + Exercise	36.46 ± 3.88***,****	36.09 ± 3.02***,*****	10.52 ± 0.83	6.82 ± 0.33	5.0	7.82
HFD + SD + Exercise	11.81 ± 1.17*****,******	18.98 ± 2.13**,****,******	4.28 ± 0.41	4.05 ± 0.42	0.55	0.05
HFD + Antiox + Exercise	16.87 ± 2.59**,******	17.44 ± 3.29*,*****,******	5.35 ± 0.77	3.70 ± 0.56	0.06	0.1

Abbreviations: antiox, antioxidant; HFD, high‐fat diet; SD, standard diet.

* *p* < 0.05 respect to control group.

** *p* < 0.01 respect to control group.

*** *p* < 0.001 respect to control group.

**** *p* < 0.01 respect to HFD group.

***** *p* < 0.001 respect to HFD group.

****** *p* < 0.001 respect to HFD + Exercise group.

**FIGURE 5:**
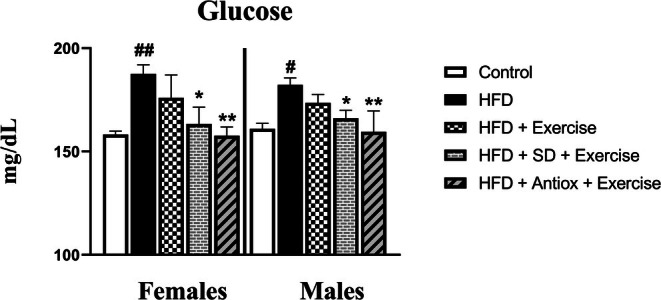
Plasma glucose levels in both female and male rats at sacrifice time (*n* = 7/sex and group): Control, high‐fat diet (HFD), HFD and exercise, and HFD groups with changes to standard (SD) or antioxidant‐rich (Antiox) diets and exercise performance. Data represent mean ± S.E.M. One‐way ANOVA with LSD post hoc. # *p* < 0.05 and ## *p* < 0.01 with respect to the control group; *p < 0.05, **p < 0.01 when compared to the HFD group.

Figure 5 shows the plasma glucose concentration at the end of the procedure in the different studied groups for both sexes. The HFD group presented the highest values, with statistically significant differences compared to the control and the two groups with modified diets and exercise in both sexes (*p* < 0.05 and *p* < 0.01 with respect to the HFD group). No differences were found between HFD and HFD + exercise in either of the sexes and groups.

**FIGURE 6:**
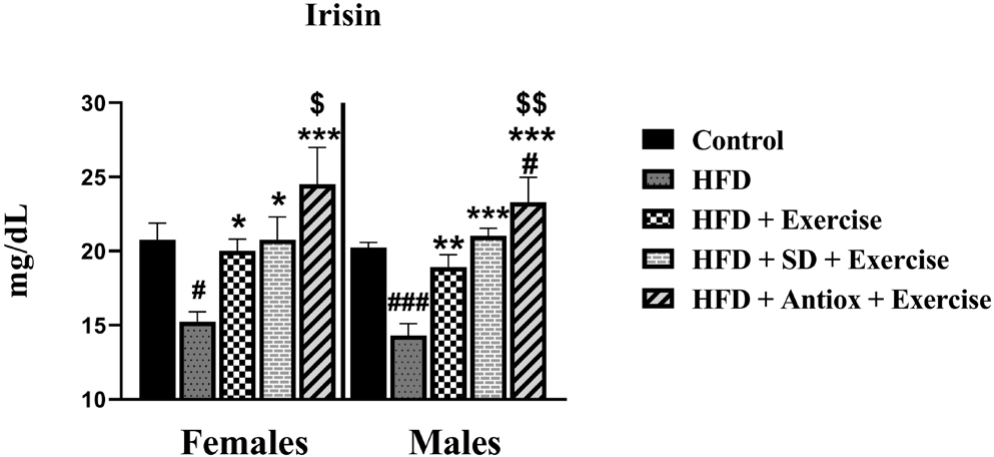
Irisin concentrations in plasma were measured in female and male rats at sacrifice time (*n* = 7/sex and group) across four groups: Control, high‐fat diet (HFD), HFD with exercise, and HFD with exercise and either a standard (SD) or antioxidant‐rich (Antiox) diet. Data represent mean ± S.E.M. One‐way ANOVA followed by LSD post hoc tests. # *p* < 0.05 and ### *p* < 0.001 with respect to the control group; *p < 0.05, **p < 0.01, and ***p < 0.001 with respect to the HFD group; $ *p* < 0.05 and $$ *p* < 0.01 with respect to the HFD + Exercise group.

Plasma irisin concentrations (Figure 6) were significantly lower in the HFD group when compared to all other groups in both sexes (*p* < 0.05, *p* < 0.01, *p* < 0.001). Moreover, the HFD + Exercise group had lower irisin values than the HFD + Antiox + Exercise group in both females (*p* < 0.05) and males (*p* < 0.01). Furthermore, the HFD + Antiox + Exercise group exhibited the highest irisin levels in both sexes, with significant differences compared to the control group in males.

**FIGURE 7:**
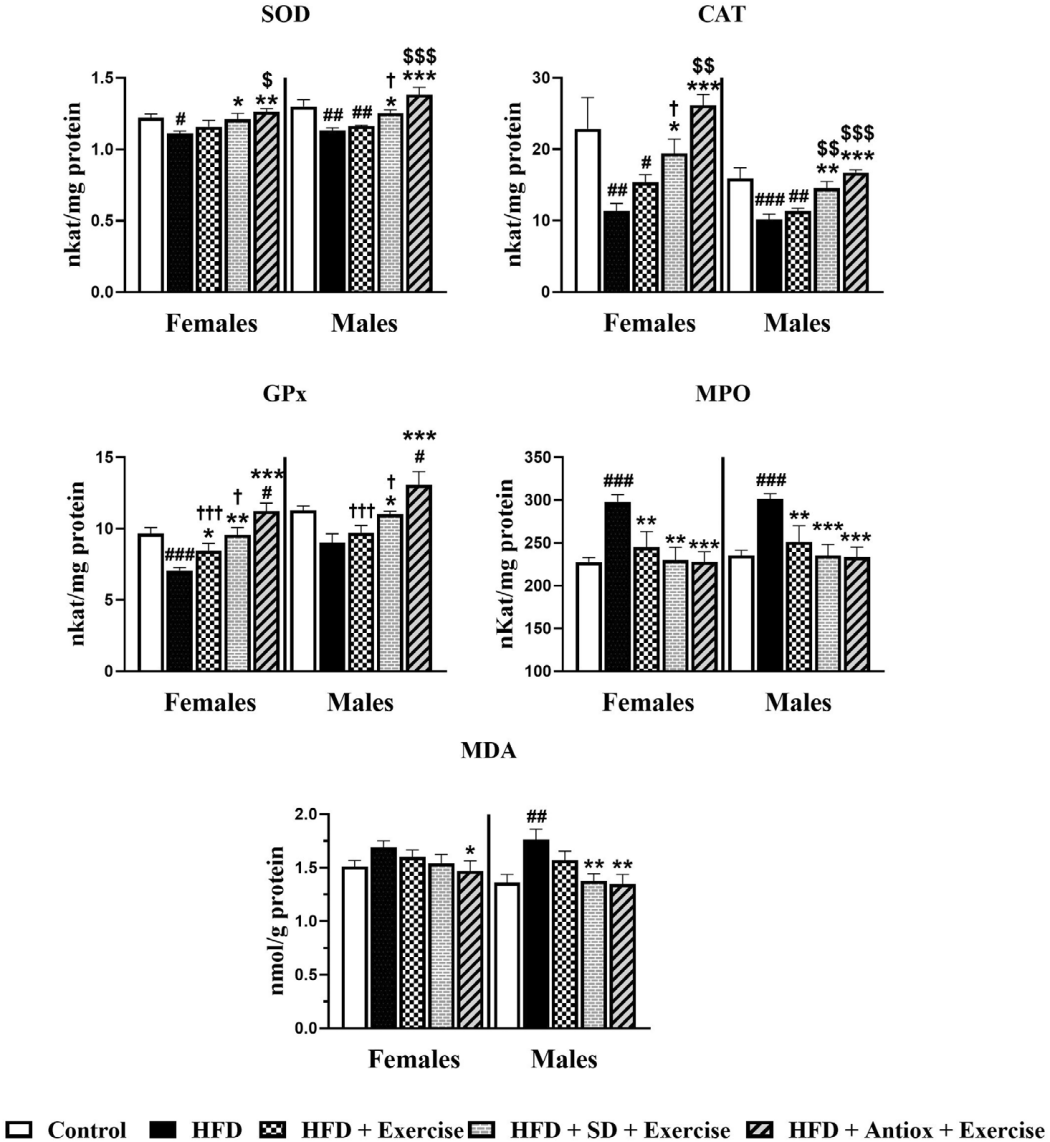
Hepatic activities of superoxide dismutase (SOD), catalase (CAT), glutathione peroxidase (GPx), myeloperoxidase (MPO) and malondialdehyde (MDA) levels in both female and male rats (*n* = 7/sex and group) for the groups control, high‐fat diet (HFD), and HFD with change to standard (SD) or antioxidant‐rich (Antiox) diets and exercise performance. One‐way ANOVA and LSD post hoc. # *p* < 0.05, ## *p* < 0.01, and ### *p* < 0.001 respect to control group; *p < 0.05, **p < 0.01, and ***p < 0.001 respect to HFD group; $$ *p* < 0.01 and $$$ *p* < 0.001 respect to HFD + Exercise group; † *p* < 0.05, ††† *p* < 0.001 respect to HFD + Antiox + Exercise group.

The observed changes in the antioxidant enzyme activities and MDA levels in the liver are represented in Figure 7. SOD and CAT activities were lower in the HFD group for both sexes compared to the control and the two groups with the combination of exercise and dietary interventions (*p* < 0.05 for SD and *p* < 0.001 for Antiox). Additionally, in females, significant differences were observed between the HFD + Exercise and HFD + Antiox + Exercise groups (*p* < 0.05 for SOD and *p* < 0.001 for CAT). In males, for SOD the HFD + Exercise group showed significant differences compared to the control and HFD + Antiox + Exercise groups (*p* < 0.01 and *p* < 0.001). For CAT, similar differences were also observed (HFD + Exercise vs control (*p* < 0.01) and vs HFD + Antiox + Exercise (*p* < 0.001)) but also with respect to the HFD + SD + Exercise group (*p* < 0.01). GPx activity also showed lower values for the HFD group compared to all the groups in females (*p* < 0.05, *p* < 0.01, and *p* < 0.001), and all groups except HFD + Exercise in males (*p* < 0.05 and *p* < 0.001). Additionally, in both sexes, the HFD + Antiox + Exercise group had higher values, showing statistically significant differences compared to the control, HFD + Exercise, and HFD + SD + Exercise groups (*p* < 0.01 and *p* < 0.001). MPO activity was higher in the HFD group compared to all the other groups in both sexes (*p* < 0.01 and *p* < 0.001) and no differences were found between the other interventional groups. Lastly, MDA levels were higher in the HFD group than in the other groups, with statistically significant differences compared to the HFD + Antiox + Exercise group in females (*p* < 0.05), and in males with all groups except for HFD + Exercise (*p* < 0.01).

**FIGURE 8:**
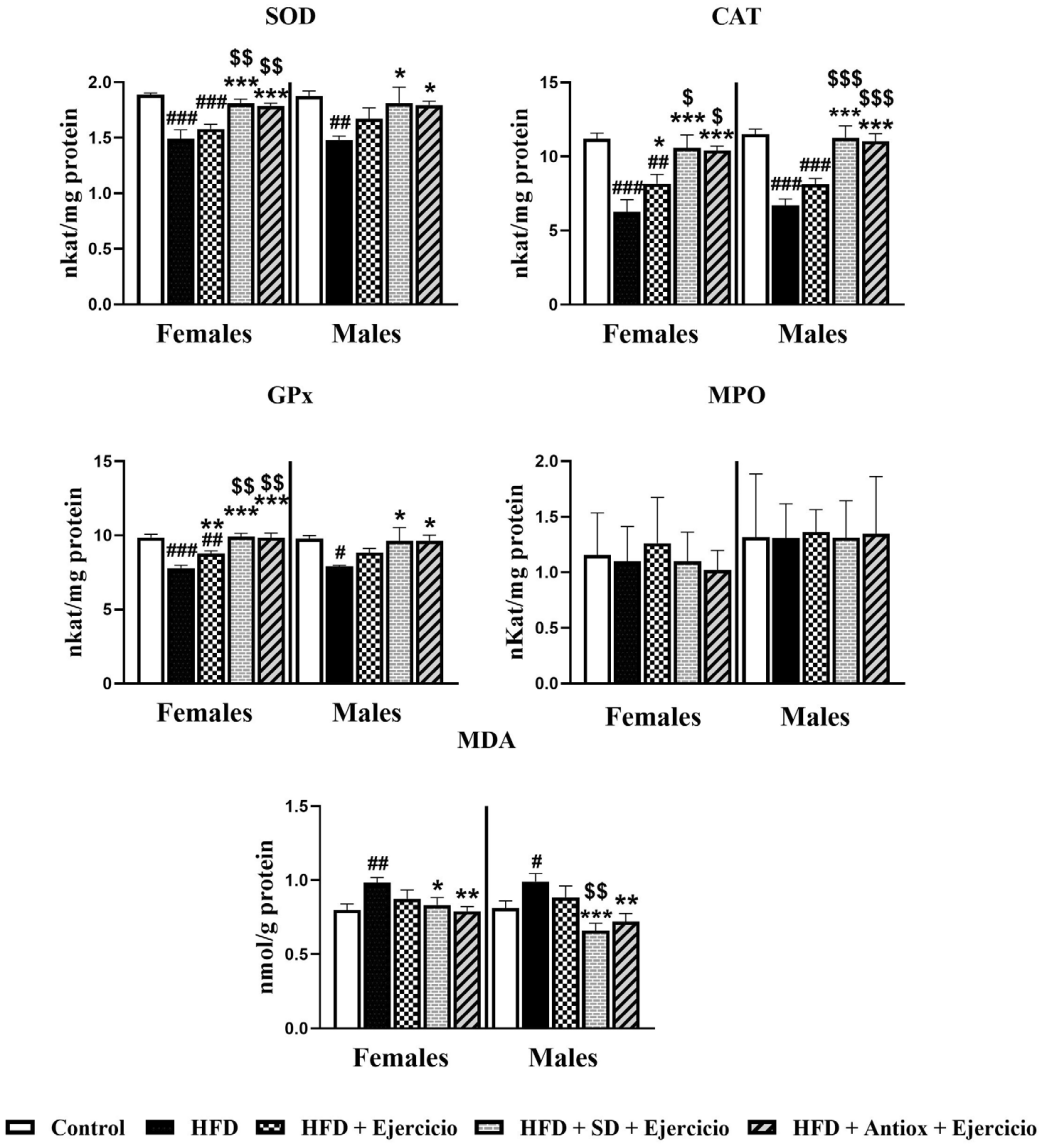
Superoxide dismutase (SOD), Catalase (CAT), glutathione peroxidase (GPx), myeloperoxidase (MPO) activities, and malondialdehyde (MDA) levels from the gastrocnemius muscle in both female and male rats (*n* = 7) for control, high‐fat diet (HFD), HFD and exercise, and HFD groups with changes to standard (SD) or antioxidant‐rich (Antiox) diets combined with exercise performance. One‐way ANOVA and LSD post hoc. # *p* < 0.05, ## *p* < 0.01, and ### *p* < 0.001 with respect to control group; *p < 0.05, **p < 0.01, and ***p < 0.001 with respect to HFD group; $ *p* < 0.05 $$ *p* < 0.01, and $$$ *p* < 0.001 with respect to HFD + Exercise group.

In the case of gastrocnemius muscle (Figure 8), the results are similar to those observed in the liver. For SOD activity, significant differences were found for the HFD group compared to all other groups, except the HFD + Exercise group, in both sexes (*p* < 0.05, *p* < 0.01, and *p* < 0.001). Additionally, in females, the HFD + Exercise group showed significantly lower values than the other three groups (*p* < 0.01 and p < 0.001). CAT activity also showed lower values for both HFD and HFD + Exercise, with significant differences compared to the other three groups in both females and males (when compared to HFD: *p* < 0.05 and p < 0.001 with respect to only performing exercise in females and to the combination of diet and exercise in both sexes; for HFD + Exercise *p* < 0.05, *p* < 0.01, and p < 0.001 compared to the rest of the groups also in both sexes). Regarding GPx activity, a similar pattern observed for CAT activity was observed. For females, the HFD group showed significant differences compared to all other groups (*p* < 0.01 and *p* < 0.001), while in males, significant differences were found with all groups (*p* < 0.05) except with HFD + Exercise. Importantly, in females, the values for HFD + Exercise were sufficiently low to exhibit statistically significant differences compared to the control (*p* < 0.01) and the two other interventional groups (*p* < 0.01). MPO activity in gastrocnemius muscle was unchanged among all the groups in both sexes without statistical differences. Lastly, MDA levels were significantly higher in the HFD group compared to the other groups in both sexes, with significant differences observed relative to the control group and the groups combining diet and exercise (*p* < 0.05, *p* < 0.01, *p* < 0.001). Additionally, significant differences were found between the HFD + Exercise and HFD + SD + Exercise groups in males (*p* < 0.01).

**FIGURE 9:**
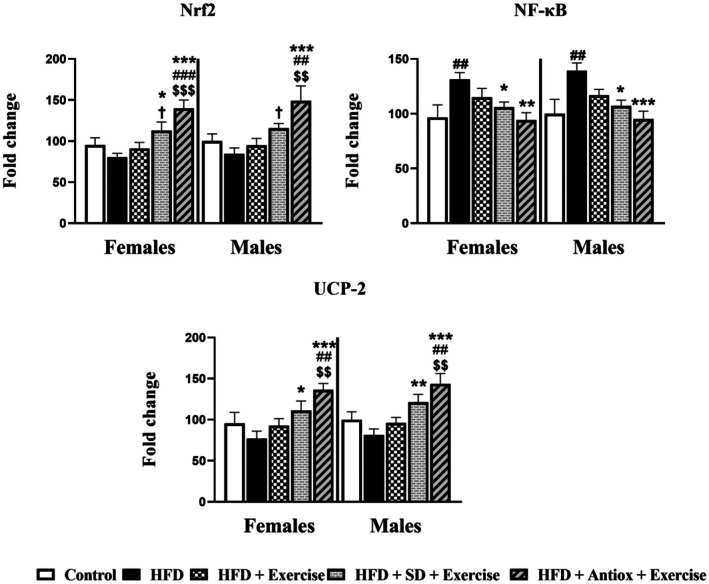
Gene expression of Nrf2, NF‐kB, and UCP‐2 in both female and male rats (*n* = 7) from the liver for the groups control, high‐fat diet (HFD), HFD and exercise, and HFD groups with changes to standard (SD) or antioxidant‐rich (Antiox) diets combined with exercise performance. One‐way ANOVA and LSD post hoc. ## *p* < 0.01 and ### *p* < 0.001 with respect to control group; *p < 0.05, **p < 0.01, and ***p < 0.001 with respect to HFD group; $$ *p* < 0.01 and $$$ *p* < 0.001 with respect to HFD + Exercise group; † *p* < 0.05 with respect to HFD + Antiox + Exercise group.

Hepatic gene expressions for both sexes are represented in Figure 9. Nrf2 expression was diminished in both sexes compared to the other groups, although the statistical differences were found when the groups changed the diet together with the exercise performance. The higher expression was for the antioxidant diet + exercise group being significant with respect to control (*p* < 0.001 for females, *p* < 0.01 for males) and the HFD group (*p* < 0.001); indeed, it was superior with respect to the standard diet together with the exercise performance (*p* < 0.05). The expression of NF‐kB in both sexes was statistically higher for the rats that fed a high‐fat diet during all the experiment with respect to the other groups, although the values recovered to the control values after the intervention with the combination of exercise with both diets (for SD, *p* < 0.05 in both sexes; for antiox *p* < 0.001 for females and *p* < 0.001 for males). UCP‐2 gene expression followed a similar response; the HFD group displayed the lowest values which were recovered after interventions, being statistically significant with respect to the combination of the exercise and SD (*p* < 0.05 for females, *p* < 0.01 for males) and antioxidant diet (*p* < 0.001).

## 4 Discussion

In the present study, a HFD significantly increased body weight and impaired motor performance in both female and male rats compared with animals that followed a standard diet, and was associated with higher plasma glucose levels and greater hepatic lipid accumulation. Moreover, the HFD induced increased levels of oxidative stress markers, as indicated by antioxidant enzymes and MPO activities and lipid damage, although females on the HFD did not show a statistically significant effect on MDA levels. Additionally, livers from both sexes presented increased expression of NF‐κB, indicating an inflammatory environment. Altogether, these results reflect metabolic alterations commonly associated with excessive hepatic lipid accumulation and inflammation also characteristic of MAFLD (Badmus et al. 2022). Indeed, fat accumulation in the liver leads to steatosis, which can progress to more advanced stages such as steatohepatitis, and this process results in increased hepatic inflammation and cellular damage (Altajar and Baffy 2020). Previous studies have reported that feeding mice a 60% lard‐based diet for 18–20 weeks can lead to substantial lipid accumulation and more advanced liver pathology, such as steatohepatitis (Sardi et al. 2021). The present results are consistent with this evidence, although in our model the experimental design aimed to induce steatosis without progressing to advanced stages Not only the liver but also other tissues may be affected. The interaction of excessive calorie intake, lack of physical activity, and genetic predisposition disrupts the regulatory mechanisms of energy metabolism, connecting adipose tissue, skeletal muscle, and the liver. This indicates a close relationship between these tissues, given that lipid uptake and lipid droplet formation also rise in muscle tissue (Altajar and Baffy 2020). Furthermore, there is an increase in proteolysis and muscle degradation, along with alterations in the production of proteins such as irisin, which are related to increased body energy expenditure. Moreover, lipid accumulation in muscle induces a reduction in glucose uptake, making it less efficient at using glucose as an energy source (Altajar and Baffy 2020). In the present work, at the three‐month point, both female and male HFD groups exhibited the worst motor coordination results, in line with previous studies (Kumar et al. 2023; Singh et al. 2021).

The introduction of a physical exercise protocol for two months reduced the hepatic lipid content even though the weight was similar to the HFD group. However, the combination of a healthier diet and physical exercise over a two‐month period reduced body weight gain and hepatic lipid accumulation. Introducing exercise also resulted in a marked enhancement of motor coordination in the males; and the implementation of dietary changes and exercise over the two months led to improved motor performance in both sexes compared to their baseline values. In other studies, it was observed that coordination in rats can be improved with dietary changes, although these studies were based on plant extracts or nutraceutical formulations together with a HFD (Bhandari et al. 2022; Gabbia et al. 2022). In the present work, the HFD used for both sexes was replaced with a healthier alternative, including polyphenol‐rich foods, such as vegetables and fruits, to mimic previous reports showing that polyphenols can enhance synaptic plasticity and reduce oxidative stress (Gabbia et al. 2022).

Regarding plasma glucose levels, the combined intervention was able to reduce them, as observed in previous studies. For instance, rats subjected to HFD for extended periods not only exhibited glucose intolerance but also impairments in insulin homeostasis (Auberval et al. 2014; Lozano et al. 2016). However, swimming for 1 h reduced glucose levels in a model of HFD‐fed male rats (Al‐Thepyani et al. 2022). Furthermore, HFD induces systemic inflammation and oxidative stress, factors that contribute to the development of insulin resistance, and overall metabolic dysfunction (Tan and Norhaizan 2019). The effect of exercise in these models has also been shown in previous works to improve insulin sensitivity, which translates into a reduction in plasma glucose levels and better management of carbohydrate metabolism (Al‐Thepyani et al. 2022). Not only physical exercise but also its combination with antioxidant compounds has been shown to have a positive effect. In 2023, a study reported the benefits of aerobic exercise and vitamin E in male rats with NAFLD. The study revealed that exercise combined with vitamin E promoted an increase in the hepatic AMPK pathway, which is closely related to lipid metabolism, a reduction in hepatic oxidative stress, and improved levels of lipid metabolism indicators (alanine transaminase, aspartate transaminase, and triglycerides) in plasma (Bai et al. 2023). Another study concluded that swimming exercise combined with silymarin and vitamin C in male Wistar rats had beneficial effects on hepatic inflammation and oxidative stress. This study demonstrated a reduction in TNF‐α/NF‐κβ gene expression, as well as an increase in PPAR mRNA expression in the liver (Aghaei et al. 2023). These findings are consistent with the results obtained in the current work including not only males but also females, highlighting the importance of the combination of physical activity and dietary changes as an intervention to mitigate the adverse effects of an HFD. This synergistic effect observed in the current work is also supported by the observed irisin changes in plasma. It has been described that myocyte contraction can induce the release of different myokines, helping communication between tissues. These myokines act as endocrine mediators, exerting their effects mainly through transmembrane and nuclear receptors (Giudice and Taylor 2017). Among them, irisin has the ability to maintain metabolic homeostasis and reduce the pro‐inflammatory status (Zhao et al. 2022). This myokine stimulates the capacity to convert white fat into brown fat, promoting enhanced lipid metabolism in adipose tissue (Ma et al. 2021). Additionally, it helps improve insulin sensitivity, facilitating glucose utilization as energy, and improving fatty acid oxidation (Liu et al. 2022). A HFD has been shown to decrease irisin levels in male mice muscle when compared to a chow diet (Lu et al. 2016; Wang et al. 2023). The low‐grade chronic inflammation induced by HFD inhibits irisin production by interfering with cellular signaling pathways involved in its synthesis, such as fibronectin type III domain‐containing protein 5 (FNDC5), the precursor of irisin (Li et al. 2021). The results of the present experiment also showed this decrease in irisin levels in the HFD groups of both sexes, suggesting a common pathway independent of sex. When exercise was combined with an HFD or a healthier diet, plasma irisin levels were restored, especially in the antioxidant‐enriched group. This could be related to the improvements also observed in the liver, since this myokine exerts antioxidant and anti‐inflammatory properties (Singh et al. 2021; Zhao et al. 2022) and is capable of reducing lipid accumulation, and suppressing lipogenesis and gluconeogenesis (Liu et al. 2015). Additionally, irisin has been described to lessen oxidative stress through activation of the Nrf2 pathway in vitro (Mazur‐Bialy and Pochec 2021) and suppressing ROS production through upregulation of the mitochondrial UCP‐2 (Bi et al. 2019), among others. In the current work, increased expression of Nrf2 and UCP‐2 in the liver was observed, which could be related to the antioxidant activity of irisin. Different works in vivo have also observed the antioxidant effects of irisin via a rise in Nrf2 activity in a model of hepatic fibrosis (Shi et al. 2020) and a NASH model (Chen et al. 2019). Finally, irisin has been shown to inhibit inflammation by reducing the activity of the NF‐κB pathway (El‐Kot et al. 2023; Shen et al. 2019); this is consistent with the return to control levels of hepatic NF‐kB gene expression observed after diet reversal in combination with exercise in the current work.

Regarding oxidative stress, the results obtained in both the liver and gastrocnemius muscle corroborate the overall physiological conditions suggested by other analyses across the different experimental groups. The lowest values of the antioxidant enzyme activities were observed in the HFD group, suggesting that this diet impairs the ability to neutralize ROS in both tissues, potentially leading to cumulative oxidative damage in hepatocytes and muscle cells (Mazzoli et al. 2019; Zouaoui et al. 2021). When exercise was included in the group with a continuous HFD throughout the entire experiment, the antioxidant capability improved, but not enough to reach the control values for all the biomarkers. This indicates that while exercise partially enhances hepatic and muscle antioxidant capacities, it may have been insufficient to fully counteract the damage caused by hepatic lipid deposits (Wang et al. 2022). In line with this, the animals following a HFD showed the highest lipid damage in the liver and muscle that was moderately reversed with exercise in both sexes. However, in the liver, greater differences were observed in males in which the dietary modifications together with exercise performance allowed them to reach MDA control values. In females, the differences were only observed between the HFD group and the HFD + Antioxidants + Exercise group. This differential finding may be attributed to the effect of estrogens in females, as these hormones have been shown to play a role in lipid regulation, modulating detrimental effects in the liver (Yang et al. 2021), and clinical studies suggest that both sex and age could be determining factors for plasma MDA levels (Pinchuk et al. 2019). Thus, diet and exercise induced similar activity levels of antioxidant enzymes compared with controls, demonstrating an enhanced ability to neutralize ROS when both factors were combined in both sexes. This is particularly evident in dietary interventions involving antioxidant supplements, which have shown effectiveness in reducing lipid peroxidation and inflammatory markers (Lima Rocha et al. 2022; Sun et al. 2019; Zouaoui et al. 2021). Studies using a diabetic state (Kumar et al. 2023) and rats fed an HFD for 12 weeks (Eleazu et al. 2021) observed a reduction in the antioxidant capacity of muscle cells by measuring SOD, CAT, and GPX. To further support the idea of increased oxidative stress, they also found that the lipid damage in muscle significantly increased in the HFD group. The previous results agree with the current ones, in which the same differences for antioxidant ability and lipid damage were observed for both sexes in the gastrocnemius muscle. In fact, it has been described that a HFD also promotes increased lipid peroxidation in the muscle tissue of obese male rats (Schaalan et al. 2018). The inclusion of antioxidant compounds in the diet, along with regular exercise, has also been shown to reduce MDA levels in the muscle of male rats (Schaalan et al. 2018; Termkwancharoen et al. 2022), and myokines such as irisin could help improve hepatic status as they enhance fatty acid oxidation (Altajar and Baffy 2020) due to their antioxidant and anti‐inflammatory properties (Zhao et al. 2022). The previous results have focused on one sex, but in the present study, it was observed that this lipid damage caused by HFD can be reversed through the combination of exercise and diet in both female and male rats. Although MPO activity was unchanged in the muscle, probably due to the moderate exercise performed by the rats, this activity in the liver was significantly higher in the HFD group. MPO, a marker of inflammation and oxidative stress, highlights the pro‐inflammatory environment fostered by HFD. In this sense, it has been shown that pharmacological inhibition of MPO prevented and reversed HFD‐induced insulin resistance in male mice (Piek et al. 2019). Additionally, MPO‐deficient female mice have shown a lower accumulation of fat and an attenuation of inflammation in tissues such as the liver and adipose tissue, compared to the same animals fed an HFD (Rensen et al. 2012). However, dietary modifications and physical exercise may help reduce these levels (Albrahim and Alonazi 2021; Lasker et al. 2019). In the present study, it was observed that both exercise alone and in combination with dietary modifications may contribute to the reduction of MPO levels comparable to the control in both sexes.

One limitation of the current work relates to the HFD, as this was not given ad libitum throughout the entire experiment, which does not fully reflect human dietary behavior. However, this was chosen to avoid excessive weight gain and advanced stages of liver disease, such as severe steatosis or steatohepatitis, which may arise with prolonged unrestricted access to HFD. The primary aim was to induce hepatic steatosis without progressing to more severe pathological stages, although future studies should be focused on an advanced state of the pathology to assess the effects of the combination of exercise and diet. In addition, the moderate exercise that the animals performed in the current work was not expected to induce inflammatory changes in the muscle at a level sufficient to observe statistical differences; however, MPO levels will be important after exhaustive exercise, a factor that should be addressed in future studies.

In conclusion, this study shows that prolonged exposure to a HFD leads to marked hepatic lipid accumulation, increased oxidative stress in both liver and muscle, and impaired motor coordination in female and male rats. These alterations were accompanied by decreased antioxidant enzyme activity and greater lipid peroxidation, confirming the detrimental effects of this dietary pattern. Importantly, the obtained findings demonstrate that the combination of dietary modifications and exercise was more effective than either intervention alone, partially restoring antioxidant defenses, reducing lipid damage, and improving functional outcomes. The addition of an antioxidant‐enriched diet further enhanced these effects, particularly in reducing oxidative and inflammatory biomarkers, suggesting a synergistic effect when combined with exercise. Sex‐specific responses were also observed and, although both sexes benefited from combined interventions, males exhibited greater improvements in hepatic lipid damage with exercise, whereas females showed enhanced motor coordination when dietary modifications and exercise were combined. Overall, these findings emphasize the central role of lipid accumulation and oxidative stress in HFD‐related alterations, and support the use of combined lifestyle interventions as effective strategies to mitigate these alterations. Future research should address sex‐specific mechanisms in greater depth to better refine prevention and treatment approaches.

## Conflicts of Interest

The authors declare no conflicts of interest.

## Supporting information


**Figure S1:** cph470074‐sup‐0001‐FigureS1.jpg.

## Data Availability

The data that support the findings of this study are available from the corresponding author upon reasonable request.
