# Supplementary figures and images for: Effects of Combining Exercise and Dietary Shifts on Motor Coordination and Oxidative Markers in a High‐Fat Diet Model in Rats
Source: Compr Physiol. 2025 Nov 25;15(6):e70074. doi: 10.1002/cph4.70074 (PMC12647931; doi:10.1002/cph4.70074)

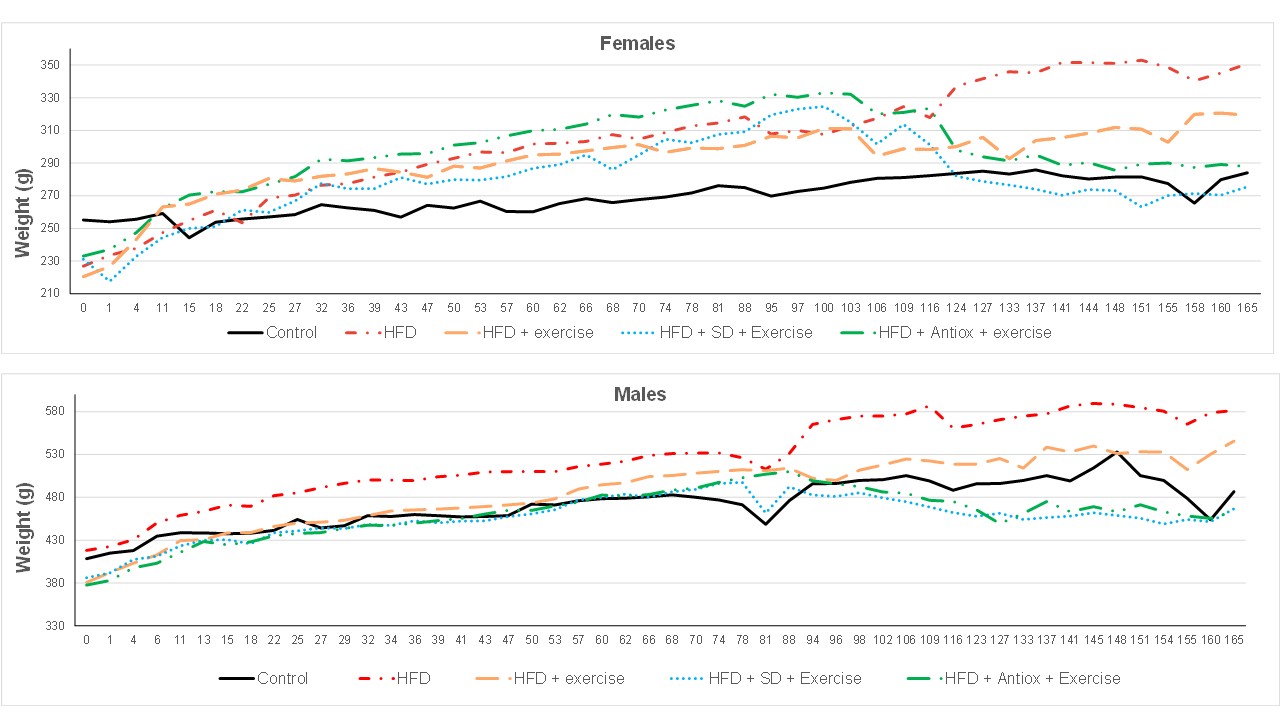

Supplement: Supplementary file 1 — Figure S1: cph470074‐sup‐0001‐FigureS1.jpg. [file CPH4-15-e70074-s001.jpg]
